# The Wonder Approach to learning

**DOI:** 10.3389/fnhum.2014.00764

**Published:** 2014-10-06

**Authors:** Catherine L’Ecuyer

**Affiliations:** Educational Consultant, Educar en el Asombro™Barcelona, Spain

**Keywords:** Wonder Approach, learning, attachment, sensitivity, beauty, behaviorism, reality-based consciousness, reality deficit

## Abstract

Wonder, innate in the child, is an inner desire to learn that awaits reality in order to be awakened. Wonder is at the origin of *reality-based consciousness*, thus of learning. The scope of wonder, which occurs at a metaphysical level, is greater than that of curiosity. Unfortunate misinterpretations of neuroscience have led to false brain-based ideas in the field of education, all of these based on the scientifically wrong assumption that children’s learning depends on an enriched environment. These beliefs have re-enforced the Behaviorist Approach to education and to parenting and have contributed to deadening our children’s sense of wonder. We suggest wonder as the center of all motivation and action in the child. Wonder is what makes life genuinely personal. Beauty is what triggers wonder. Wonder attunes to beauty through sensitivity and is unfolded by secure attachment. When wonder, beauty, sensitivity and secure attachment are present, learning is meaningful. On the contrary, when there is no volitional dimension involved (no wonder), no end or meaning (no beauty) and no trusting predisposition (secure attachment), the rigid and limiting mechanical process of so-called learning through mere repetition become a deadening and alienating routine. This could be described as training, not as learning, because it does not contemplate the human being as a whole.

*Omnes homines natura scire desiderant*.

All men by nature desire to know. (Aristotle)

## Introduction

It is well documented that the organic constitution of a child’s brain plays a key role in his development. But how does a child learn? Is the organic structure of the brain what drives the child to learn? Or is there any state of mind emerging from the brain that is responsible for the desire to learn? Or is the child’s learning the mere result of mechanical responses to external stimulus? What is the difference between a child that seizes learning opportunities and one that does not under the same external conditions? Throughout the last decades, many neuroscientists have tried to understand the sense of self, of consciousness, in most cases recognizing that the issue escapes the scope of neuroscience. As a matter of fact, Huxley said, “how it is that any thing so remarkable as a state of consciousness comes about as the result of irritating nervous tissue, is just as unaccountable as the appearance of the Djin when Aladdin rubbed his lamp” (Huxley and Youmans, 1868).

What is the relationship between self-consciousness and learning? What is the origin of learning? Does it come from within the human being, or from without? It is organic, or intangible? Is it a by-product of the neurological makeup, or does it lie deeper than the brain?

Dan Siegel, who himself recognized that “the idea of intention is itself a philosophical puzzle” (Siegel, 2012), also said:

“When we think about psychological development, about the developing mind, it is helpful to think about what the “psyche” actually is. There is an entity called the psyche or the mind that is as real as the brain, the heart, or the lungs, although it cannot be seen directly with or without the aid of microscopes or other tools of modern technology. One definition of the psyche is: “(1) the human soul; (2) the intellect; (3) psychiatry—the mind considered as a subjectively perceived, functional entity, based ultimately upon physical processes but with complex processes of its own: it governs the total organism and its interaction with the environment” (Webster, 1996). Within this definition, we can see the central importance of understanding the psyche, the soul, the intellect, and the mind in understanding human development” (Siegel, 2001).

It is not a coincidence that world spiritual leaders took interest in Siegel’s Interpersonal Neurobiology. In 1999, John Paul II invited Siegel to deliver a speech (Towards a Biology of Compassion: Relationships, the Brain and the Development of Mindsight Across the Lifespan) at the Vatican; in 2009, the Dalai Lama shared a panel with Siegel on the scientific basis of compassion.

Regardless of whether we hold religious beliefs or not, and of what they are, there is a growing sense that the motor of the human being is something intangible that cannot be seen with the eye nor can be measured with scientific instruments. Does it emerge from the brain, from interpersonal interaction (as suggested by Siegel’s Interpersonal Neurobiology), is it previous to any other human process, or is it embodied within the brain? At this point, a multidisciplinary approach is necessary in order to get a broader picture.

## Wonder: a *reality-based consciousness* approach to learning

More than three centuries B.C., the Greek philosophers Plato and Aristotle said that the principle of philosophy was wonder (Aristotle, 2014; Plato, 2014b), the first manifestation of something intangible that moved the human being towards reality, also defined by Aquinas as “the desire to learn” and later by the English philosopher Francis Bacon as “the seed of knowledge”. Chesterton talked about wonder as a principle, not a consequence: “This elementary wonder, however, is not a mere fancy derived from fairy tales; on the contrary, all the fire of fairy tales is derived from this” (Chesterton, 2004a).

More recent authors have written on the importance of wonder for the purpose of awakening ecological awareness in the child (Carson, 1965), as pedagogical proposals or tools to be used in the classroom (Legrand, 1960; Lipman and Sharp, 1986; Egan et al., 2013). But to this day, and despite the fact that it has been discussed during more than twenty-four centuries, wonder has not yet been proposed as a theory of learning.

Not only is the idea of wonder as old as Greek philosophy, it is also a universal phenomenon, well-known by any parent. *Why is it not raining upwards? Why is the moon round and not square?* Children have asked these questions since the beginning of time. When children ask these questions, they might not be demanding an answer. Rather, they might be wondering in the face of reality. They are wondering because it rains downwards and because the moon is round. When children ask these questions, they are, as Plato and Aristotle suggested, philosophizing. They are surprised at the mere fact of seeing that things “are”. Babies wonder when they first see the sky, the stars, the face of their mother, when they first touch the grass, see a shadow, experience gravity and so on. As Chesterton wrote: “The most unfathomable schools and sages have never attained to the gravity which dwells in the eyes of a baby of 3 months old. It is the gravity of astonishment at the universe, and astonishment at the universe is not mysticism, but a transcendent common sense. The fascination of children lies in this: that with each of them all things are remade, and the universe is put again upon its trial. As we walk the streets and see below us those delightful bulbous heads, three times too big for the body, which mark these human mushrooms, we ought always to remember that within every one of these heads there is a new universe, as new as it was on the seventh day of creation. In each of those orbs there is a new system of stars, new grass, new cities, a new sea” (Chesterton, 2005).

## The scope of wonder

The scope of wonder, as discussed in this present article, is greater than a mere emotional response. It is worth mentioning that many authors, a detailed analysis of which may be found in Artemenko (1972), have referred to “étonnement” (an alternative French translation for “wonder”) as a spectrum of emotions ranging from a reaction to novelty, to fear, to surprise, etc. According to the Wonder Approach discussed in this article, the emotional response would be a possible consequence of wonder, not wonder as such.

Furthermore, the scope of wonder goes beyond curiosity. Curiosity is the urge to explain the unexpected (Piaget, 1969), or the urge to know more (Engel, 2011), and may be an instinctual response. Wonder is the desire to know the unknown, as well as the already known. Before the already known, a child may wonder again and again, because to wonder consists in “never taking anything for granted”, even that which is already known. So regardless of whether a thing is already known, the wondering attitude is to consider this thing “as if for the first time”, as well as “as if for the last time”. This metaphysical manner of thinking is typical of a person that realizes that the world is, but also, that could not have been at all. We are—the world is—contingent. If we cease to exist, the world still exists… We participate in something greater than us, the world that surrounds us. Wonder is precisely what allows us to be conscious of the surrounding reality, through humility and gratitude. Wonder is a sort of *reality-based consciousness*, which perhaps could shed some light on the issue of the subjective aspect of experience that is part of what some have called the “hard problem of consciousness” (Chalmers, 1995).

## The wonder approach vs. the behaviorist approach to education

Contrary to the Wonder Approach would be the Behaviorist Approach to education, according to which everything is programmable and the volitional aspect is irrelevant because the child is completely dependent on the environment in order to learn. Therefore, according to this view, education would be reduced to “bombarding with information” (the more the better) and to “training in habits” (as mere mechanical repetition of actions), as reflected in John Watson’s promise “Give me a dozen healthy infants, well-formed, and my own specified world to bring them up in and I’ll guarantee to take any one at random and train him to become any type of specialist I might select…” (Watson, 1930). The Behaviorist Approach emphasizes the accumulation of information (knowledge), on external behaviors (skills and mechanical habits) and their emotional and physical reactions in given situations, rather than on the person’s internal mental states, such as intentionality, which are much more complex.

According to the Wonder Approach, learning would start from within; it would be an inner personal “desire”. The environment would be important, but the environment would not be *per se* what makes the child learn. And so it follows that “more” would not necessarily be better.

In recent years, neuroscience has come to the conclusion that more is not necessarily better and that learning is not a matter of overwhelming “enrichment” or excessive intellectual stimulation:

“There is no need to bombard infants or young children (or possibly anyone) with excessive sensory stimulation in hopes of “building better brains”. This is an unfortunate misinterpretation of the neurobiological literature—that somehow “more is better”. It just is not so. Parents and other caregivers can “relax” and stop worrying about providing huge amounts of sensory bombardment for their children. This synaptic overproduction during the early years of life has been proposed to allow for a likelihood that the brain will develop properly within the “average” environment that will supply the necessary minimal amount of sensory stimulation to maintain necessary portions of this genetically created and highly dense synaptic circuitry” (Siegel, 2001).

The “unfortunate misinterpretation of the neurobiological literature” has brought on a series of “neuromyths” and false beliefs in the field of education, such as “more is better” and “earlier is better” (American Academy of Pediatrics, 1968; Goswami, 2006; Howard-Jones, 2007; Hyatt, 2007). These unfortunate misinterpretations have also encouraged false brain-based ideas in the education industry, with products such as Brain Gym^®^, Baby Einstein™, the use of flashcards in classrooms, attempt to repattern the child’s brain through co-ordination exercises, so-called educational toys and videos, etc., all of these based on the scientifically wrong assumption that children’s learning depends on an enriched environment during the period of synaptogenesis. Valuable time and money, both of which schools often lack, is being spent in obeisance to these myths (Howard-Jones, 2009). These beliefs have re-enforced the Behaviorist Approach to education and to parenting and have contributed to deadening our children’s sense of wonder. The process by which this is suggested to have happened is explained below in more detail.

## Beauty triggers wonder in the child

Children wonder because they realize that a thing “is”, while it could “not be”. What is it in the “being” of the things that surround children that trigger wonder in them? The Greek philosophers have identified some of the properties of “being”, one of which is beauty. Thus, one of the properties of “being” of a thing that triggers wonder in children is beauty.

What is beauty? Does it always relate to personal taste? The beauty that philosophers refer to is not a mere esthetic beauty that depends on fashion and tastes and that usually triggers a desire for possession. The beauty to which philosophers, such as Aristotle, Plato and Aquinas refer is defined as the visible expression of truth and goodness. That is why Plato writes: “the power of the good has retired into the region of the beautiful” (Plato, 2014a). In the 21st Century, the distinction between metaphysical and cosmetic beauty might be better understood by reflecting on Dove’s commercial slogan “Talk to your daughter about beauty before the beauty industry talks to your daughter”.

So what would be beautiful to a child? If beauty is the visible expression of truth and goodness, beauty for a child is anything that responds to the truth and goodness of childhood. For example, children are innocent, they learn at a slower pace compared with adults, they need to trust in an attachment figure as we shall see below, they learn from within, they need silence to process information, they have a special affinity with the natural world and with mystery (a mystery is an infinite opportunity to know, which would be expected to awaken wonder, a desire to know), and so on. A beautiful environment is one that triggers wonder, which results in learning. An environment that respect a child’s pace and his innocence, an educational content that goes beyond the rational and mechanical explanation of things and that leaves some space for mystery, opportunities for silence and contemplation, etc.

What is ugliness? Does it exist? Aquinas says that “beauty can be found in all existing beings” (Aquinas, 1965). This is because one of the properties of “being” is beauty, and so for the mere fact of “being”, all things hold beauty in themselves, although they might do so in different proportions. Thus, ugliness may be defined as the absence of beauty, which could be partially, but never completely absent. A thing that has a small proportion of beauty in it could be defined as “empty”, “vulgar”, “not excellent”, or “meaningless”. Ugliness means less motivation for children to wonder. Children might be fascinated, their mind might be paralyzed before an ugly thing, but it does not trigger wonder in them, it does not broaden their intellectual horizons. So a relevant question would be: what would happen to children’s learning if the educational system paid more attention to beauty and filtered what does not hold enough of it?

But how do we know what holds beauty and what does not? Is there an instrument that can measure the percentage of beauty in what surrounds us? Obviously, there is no such instrument. There are sensitive skins and elephant skins, so to speak. The parent’s and the educator’s sensitivity is what makes them able to perceive the child’s needs, what is true and good for them. It is what makes them attune to beauty. In one of the most comprehensive existing studies on child care (NICHD, 2006), a mother’s sensitivity (a mother’s responsiveness to her children’s true needs) has been considered the most consistent predictor of a child’s healthy development.

## Sensitivity is what makes wonder attune to beauty

When wonder encounters reality, it attunes to its beauty. This attunement requires the child’s sensitivity. Sensitivity could be defined as the capacity, not only to perceive a thing through the senses, but also to attune to the beauty that is in it. The child’s attunement process is a sort of focused attention, or empathy with reality, allowing him to feel the beauty that surrounds him.

An obstacle to this attunement would be, for example, a defect in the senses, which would prevent the child from grasping the essence of a thing. This defect could be organic, or it could be the result of an environment that does not recognize his innate desire for wonder. This could be, for example, the case of a child that has been bombarded with information, strongly stimulated from without, whose senses have been crowded and overwhelmed by intensive technological multitasking and/or consuming environment. As a result, the senses’ threshold of “feeling” reality goes down and wonder has less and less to expect from and to work with, until it is as though deadened. When this happens, the child becomes passive, bored and muddled and is increasingly dependant on the external environment in order to pay attention and to learn. This dependence is what would be described in the educational language as “lack of motivation”. As the threshold of “feeling” reality is dropping to dramatically low levels, the child needs more and more external stimulus in order to “feel” reality. This is when addictions could come into the picture.

This phenomenon has been considered relevant in the study of media consumption by children. Research on television viewing has established a relationship between television viewing by children under the age of three and attention problems later on in life (Christakis et al., 2004). According to the overstimulation hypothesis, “the surreal pacing and sequencing of some shows might tax the brain or parts of it, leading to short-term (or long-term) deficits” (Christakis, 2011). In Christakis’ words, “prolonged exposure to rapid image change during critical period of brain development would precondition the mind to expect high levels of stimulation and that would lead to inattention in later life” (Dimitri Christakis, on TedxRainier). In other words, the child’s mind gets conditioned to a reality that does not normally exist in real life. And so when the mind comes back to real ordinary life, everything seems extraordinarily boring, because it cannot see the beauty in ordinary life. As there is no beauty to attract them, children easily get distracted (“distraction” is the contrary of “attraction”) and thus become completely dependent on the external environment.

In another study (Overberg et al., 2012), obese subjects could identify taste qualities less precisely than children and adolescents of normal weight. The reduced taste sensitivity makes them want to consume more. Taste sensitivity is multifactorial, so learning influence, such as exaggerated taste stimuli in early childhood, could play a role. When children’s taste is over saturated, they cease to feel and so they need more food to perceive taste qualities, what could lead them to gaining more weight. Another study (Kirsh and Mounts, 2007) concluded than violent video game exposure reduces happy facial emotion recognition.

Similar conclusions have been reached in a Stanford study (Ophir et al., 2009), in which researchers looked at what heavy media multitaskers were good at, in terms of (1) capacity to filter information according to its relevancy; (2) working memory; and (3) capacity to switch efficiently from one task to the other. The study found that they were doing worst on all of these parameters. When trying to process various thoughts “at the same time”, we are not attending to all of them in parallel at the same time, but rather shifting our attention back and forth among all of them, the result being that the thoughts that we are trying to attend to “at the same time” receive less of our attention, as we need to recover our train of thought every time we switch our attention from one task to the other. This is why the Nobel Prize laureate Herbert Simon said “What information consumes is rather obvious: it consumes the attention of its recipients. Hence a wealth of information creates a poverty of attention, and a need to allocate that attention efficiently among the overabundance of information sources that might consume it” (Simon, 1971). When the external environment overwhelms our senses, wonder is inhibited and we cease to be actively involved in paying attention. We become passive and the external input “consumes our attention”, instead of us focusing on the environment. So clearly, more is not necessarily better and learning does not depend completely on the environment, but on the inner capacity to focus the attention on one thought at a time and to recognize what has meaning and what does not.

Clifford Nass, founder and director of the Communications between Humans and Interactive Media Lab, from where the study was carried out, said, “it’s very troubling. And we have not yet found something that they’re definitely better at than people who don’t multitask (…) Multitaskers love irrelevancy” (Interview in Frontline, December 3th, 2009). In reality, what might be happening is that heavy media multitaskers, violent video game players and obese people who have lost taste sensitivity, like any other human being, crave beauty and meaning. But heavy external multi-source stimulus leads to the overwhelming of the senses, which could contribute to the loss of sensitivity to beauty and meaning. This makes them incapable of recognizing beauty, and so they search for beauty at random. As their craving for beauty is not easily satisfied, they then enter into an unending circle of compulsive consumption behaviors that make them feel less and less, until they can almost appear to be like philosophical zombies.

These searches for taste, for information, for images, are searches for beauty, for meaning. And a meaningful subjective experience could be described as the result of the encounter of a subject’s wonder with beauty. It is meaningful because the human being is made, not only from a philosophical, but also from a neurological point of view, to be attracted by beauty, through wonder. This meaningful encounter between wonder and beauty could be what make a subject’s action genuinely personal.

## Wonder and beauty are what give meaning to the repetition of actions in the child

According to Montessori, children’s repetition is the secret of perfection (Montessori, 1986). But can *any* repetition lead to perfection? A routine is commonly defined as “a regular procedure, customary or prescribed” (Webster, 1983). In the educational context, the routine is often seen as necessary because it gives children a certain sense of security and order, as the children can anticipate what comes next. But what makes routine become an obstacle to the child’s development? The routine can have an alienating effect on the child when it converts itself in a mere repetition of acts (an end in itself) that have no meaning whatsoever for the child. When this happens, the child acts in a mechanical way, is not fully conscious of what he is doing because there is no meaningful end to his action, or at least the child does not see it. As a result, the volitional, cognitive and emotional dimensions of the child are not involved, the child does not interiorize what he is doing and so there is no sustainable learning. In this context, the routine is the automation of an action. Rather than being a personal subject, the child becomes an object. This is why the result of this process would be linked to rigidity and limitation, rather than to creativity and imagination. The kind of habit involved in this situation would be the result of coercion, mere inertia, training, or perhaps addiction, but not of education. As Thomas Moore said, “Education is not the piling on of learning, information, data, facts, skills, or abilities—that’s training or instruction—but is rather making visible what is hidden as a seed” (Moore, 1997). As Aquinas (1953) points out, “before the habits of virtue are completely formed, they exist in us in certain natural inclinations, which are the beginnings of the virtues. But afterwards, through practice in their actions, they are bought to their proper completion”. Virtue starts from within, not from without. In the mechanical repetition of actions, there is no real education because there is no wonder and no opportunity for beauty. Beauty is what gives the routine meaning to the child. It is what converts the routine into what Saint-Exupery called a “ritual”, “what makes one day different from the other days, one hour different from the other hours” (Saint-Exupery, 2000).

So the differential element that converts the child’s mere mechanical repetition of actions into a meaningful ritual is beauty. This is why Montessori had children repeating what she called “practical life exercises” (she insisted that their aim was not “practical”, rather the emphasis was on the word “life”) (Standing, 1998) with “motive of perfection”. Montessori insisted on the importance of surrounding children with reality and beauty. As explained earlier, beauty is the visible expression of what is true and good for a child, of what the child’s nature is capable of possessing. How can a child’s education be the expression of truth and goodness? It is when education facilitates the child to possess that which, by his nature, he is capable of possessing. On the contrary, the education would cease to be beautiful when it does not give this opportunity to the child, or when it urges the child to possess that of which, by his nature, he is not capable of possessing. For example, a child would not be able to learn well under pressure, with high amount of external stimulus that require simultaneous thought processing, extremely fast-paced content, etc.

## Secure attachment unfolds wonder in the child

One of the well-known truths about children is that they need to develop a secure attachment relationship with their principal caregiver. How does the attachment process occur and how does it relate to wonder and beauty?

The attachment theory, first developed by Bowlby (1969) and Ainsworth (1967, 1969; Ainsworth et al., 1978), is now one of the most widely recognized and established theoretical approaches in the field of psychological development. Throughout the years, this theory has converted itself into “the dominant approach to understanding early social development” (Schaffer, 2007), has been confirmed by quantities of empirical research in psychology, neurobiology, pedagogy, psychiatry, etc., and is now being used as the basis of most social and childcare research and policy (NICHD, 2006).

According to Bowlby and to numerous studies, secure/insecure attachment is the function of the sensitivity the principal caretaker has towards the prompt resolution of an infant’s basic needs for security, safety and protection. This is why a mother’s sensitivity has been considered the most consistent predictor of a child’s healthy development. This sensitivity is responsiveness, attunement to the reality of the child, with his daily life needs. So what matters is not orchestrated enrichment inputs for children, but a million small acts of responsiveness to daily life needs. Based on the responsiveness pattern, the infant will develop an “Internal Working Model”, a paradigm that he has of himself and that will affect all of his future relationships.

For instance, if the infant receives the message: “Your needs cannot be attended to”, he will develop the Internal Working Model “I cannot trust others”, “The world is hostile”, “I am not worthy”, “I am not competent”. The result is insecure attachment. This leads the child, teenager and adult-to-be to low self-esteem, high insecurity, low social competence and resistance to exploring the unknown. *The message that the child has interiorized is that the world is hostile, that he cannot trust what is around him*. So it would be reasonable to expect that a child with insecure attachment would have a more cynical attitude towards life, one that does not easily trust in beauty, truth and goodness. Therefore, insecure attachment would be expected to inhibit a child’s capacity to perceive beauty.

On the other hand, when the infant’s basic necessities are promptly addressed, he will develop the Internal Working Model: “I can trust others”, “I am worthy”, “I am competent”. The result is secure attachment. This leads to high self-esteem and security, high social competence and interest in exploring the unknown in the child, the teenager and eventually the adult. *The message that the child has interiorized is that the world is trustworthy*. So it would be reasonable to expect that a child with secure attachment would have a greater predisposition to experience wonder, because he has a natural trusting attitude towards beauty, truth and goodness. Therefore, secure attachment would be expected to foster attunement to beauty.

Thus, one would expect the innate desire in the child for knowledge to flourish in an environment of secure attachment and to be inhibited by insecure attachment. There is a second reason for this. Once children are securely attached to their principal caregiver, they use their principal caregiver as an exploratory base to learn what is around them. What does an eight-month-old child do when introduced to a stranger? He looks at his principal caregiver, and then back and forth to the stranger, as if he were asking his caregiver for permission. What does a four-year-old child do when discovering a snail in the park? “Look mom!” This is no doubt one of the most repeated sentences in playgrounds. Children continually triangle between the reality they discover and their principal caregiver. Carson (1965) rightly points out: “If a child is to keep alive his inborn sense of wonder, he needs the companionship of at least one adult who can share it, rediscovering with him the joy, excitement, and mystery of the world we live in”. In fact, securely attached children have been found to be more intellectually curious (Arend et al., 1979). And children have been found to learn better from human interaction than from an enriched environment. For example, not only is there no relation between baby videos and word or foreign language learning, but media exposure has been associated with less vocabulary and delayed language development (Kuhl et al., 2003; Chonchaiya and Pruksananonda, 2008; Richert et al., 2010; Duch et al., 2013).

That does not mean that wonder is a by-product of secure attachment, or that secure attachment precedes wonder. On the contrary, the attachment pattern develops between around 6 months and 3 years old. It would be unreasonable to say that children under 6 month-old do not experience wonder in relation to the world. Rather, it would be reasonable to say that the attachment pattern outcome can inhibit or foster the existing potential that the child has for wonder.

If wonder is innate in the child, then it also precedes self-consciousness, which starts to appear at the age of two, when the child starts having his own biographical memory, through explicit memory (Siegel, 2012). Therefore, self-consciousness is not necessary for wonder to happen. In fact, it is notorious that infant and children have a capacity for wondering that is much greater than adults. Perhaps not having yet developed a sense of object permanence (the understanding that objects continue to exist even when they cannot be observed) has a positive effect on children’s innate sense of wonder, because they literally experience what is around them, again and again, as if it were for the first time. But object permanence cannot explain wonder, because wonder is a phenomenon that occurs throughout life.

## The triangle of wonder: the child, the attachment figure and reality

According to the Wonder Approach, the teacher is a facilitator in the process of connecting the mind, the will and the heart of the child with what is true, good and beautiful, so that when he becomes a teenager or adult, he will eventually be able to identify and discover them “by himself”.

Some interpretations of Constructivism (Piaget, 1999) suggest that the child can and should discover without any guidance. Evidence does not support educational methods such as “pure discovery without guidance” in a young learner, because if he fails to come into contact with the to-be-learned principle, discovery will not be useful in helping the learner to make sense of it (Mayer, 2004; Kirschner et al., 2006). This is because “all teaching comes from pre-existing knowledge” (Aquinas, 1953), a similar idea to what Vygotski (1978) called the *zone of proximal development*. Teaching and knowledge do not just “happen” in a magical way. The young child needs an attachment figure to mediate between him and reality, a process that some have described as *scaffolding* (Bruner, 1987; Hmelo-Silver et al., 2007).

Social Constructivism philosophy goes further by suggesting that reality is actively constructed by the child, who builds his perception through social interactions (Vygotski, 1978; Bruner, 1987). According to the Wonder Approach, neither the attachment figure nor the child can create reality ontologically speaking. Reality is prior to knowledge. As Aquinas (1953) explains, “he who teaches does not cause the truth, but knowledge of the truth, in the learner. For the propositions which are taught are true before they are known, since truth does not depend on our knowledge of it, but on the existence of things”. Beyond this ontological difference, the Wonder Approach acknowledges a subjective personal dimension (the child), as well as a social dimension to learning. However, it suggests that learning is reality-based and that *reality deficit* makes learning more difficult. In fact, it has been demonstrated that infants and children learn less from 2D images than from real face-to-face situations. This is known as the *Video Deficit Effect* (Anderson and Pempek, 2005). Furthermore, a study (Diener et al., 2008) comparing infant’s reactions to television and live events concluded that they look longer at, reach more to, show more interest in, and exhibit more fear to, real events. Also, when they were shown live and video events simultaneously, they had a preference for real events.

## Testable predictions and further investigations

Further investigation is needed to test the Wonder construct as a valid approach to learning. Our testable prediction is that wonder, beauty, sensitivity and secure attachment provide the optimal conditions for learning in children. Wonder is innate, so it is assumed to exist in infants. Beauty is understood in our context as “what responds to the truth and goodness of childhood”. Investigation is needed to define a comprehensive set of variables, although at this point in time we would expect silence, mystery, respect for a child’s pace and innocence, to be optimal conditions for wonder. Sensitivity and attachment could be measured using existing tools.

It would also be relevant to investigate whether the educator’s paradigm or anthropological mindset, namely the approach to learning used by the educator (wonder/behaviorist/constructivist/social) has more impact than the method used with the child. For example, the way flashcards are used by Montessori’s followers is different from the way they are used by Glenn Doman’s followers. We would expect the educator’s paradigm to have more impact than the educational method as such.

Finally, it would be of interest to inquire into the consequences of the loss of wonder in a child. Is the educational system promoting wonder, or inhibiting it? Why? Could the loss of wonder, incurred as a result of giving overly exaggerated importance to external stimulus in learning, shed more light on the mechanisms of the increasing number of learning problems, in which environmental factor have been said to play a role? (U.S. Department of Health and Human Services, 1999).

## Conclusion

We suggest wonder is the center of all motivation and action in the child. Wonder and beauty are what make life genuinely personal. Wonder attunes to beauty through sensitivity and is unfolded by secure attachment. When wonder, beauty, sensitivity and secure attachment are present, learning is meaningful.

On the contrary, when there is no volitional dimension involved (no wonder), no end or meaning (no beauty), no attunement between the volitional dimension and meaning (sensitivity) and no trusting predisposition (secure attachment), the rigid and limiting mechanical process of so-called learning through mere repetition becomes a deadening and alienating routine. This could be described as training, not learning, because it does not contemplate the human being as a whole.

While there is an increasing interest in an holistic and integral vision of the human being in education, there is also a tendency to conceptually fragment man into various parts and pieces, for example through theories that divide intelligence, or through the left- and right-brain balanced approach to learning, which is a consequence of an over-literal interpretation of hemisphere specialization (Goswami, 2006).

What if wonder served to bridge all of these parts and pieces in order to help make sense of them? Aristotle said, “all men by nature desire to know” (Aristotle, 2014). What if wonder were the meeting point between the volitional and the cognitive (“desire”, “to know”) dimensions of the human being? This approach involves a change in paradigm because it implies a return back to reality, a switch from self-consciousness towards *reality-based consciousness* as the starting point of learning. In the midst of multidisciplinary confusion, some have been arguing in favor of the *middleman* figure of a *neuroeducator*. Before we consider experimenting this new idea on our children, perhaps it is worth opening up the multidisciplinary debate and paying some attention to the Wonder Approach. This might well be an opportunity to re-consider the classical approach to philosophy as a relevant *middleman* between neuroscience and education. Chesterton once wrote that “the world will never starve for want of wonders; but only for want of wonder” (Chesterton, 2004b). The Wonder Approach is an attempt to prove Chesterton’s prophecy wrong, so that, in the midst of so many distractions, our children can wonder again before the irresistible beauty that surrounds them.

## Author contribution

Catherine L’Ecuyer, Bachelor of Laws, MBA, European Master in Research. She is the author of “Educar en el Asombro” (L’Ecuyer, 2012), on which is based the Wonder Approach discussed in this article.

## Conflict of interest statement

The author declares that the research was conducted in the absence of any commercial or financial relationships that could be construed as a potential conflict of interest.
